# Insight into the current practice of ototoxicity monitoring during cisplatin therapy

**DOI:** 10.1186/s40463-021-00506-0

**Published:** 2021-03-25

**Authors:** N. M. Santucci, B. Garber, R. Ivory, M. A. Kuhn, M. Stephen, D. Aizenberg

**Affiliations:** 1 Oregon Health and Science University, School of Medicine, Portland, OR USA; 2 University of California Davis Department of Otolaryngology-Head and Neck Surgery, 2521 Stockton Blvd., Sacramento, CA 95817 USA; 3 University of California Davis Medical Center, Sacramento, CA USA; 4 University of California Davis Department of Internal Medicine – Hematology/Oncology, Sacramento, CA USA

**Keywords:** Ototoxicity, Cisplatin, Hearing loss, Audiogram, Audiologic, Otologic, Monitoring Program, Quality improvement

## Abstract

**Background:**

The aim of this study is to evaluate the current state of ototoxicity monitoring for patients receiving cisplatin chemotherapy in an academic medical center with particular attention to how closely monitoring adheres to national ototoxicity guidelines.

**Methods:**

Case series including retrospective medical records review of patients (age > 18) treated with cisplatin at University of California Davis Medical Center between January 2014 and August 2017. Patient and ototoxicity related variables were analyzed. Patients that underwent a transfer of care during treatment and with less than 3 months of follow-up were excluded.

**Results:**

Three hundred seventy-nine patients met study criteria, of which 104 (27.4%) had a prior history of hearing loss. Prior to treatment, 196 (51.7%) patients were counseled regarding the ototoxic nature of cisplatin and 92 (24.3%) patients had a pretreatment audiogram. During treatment, 91 (24%) patients had documented otologic complaints. Only 17 patients (4.5%) patients had an audiogram ordered during their cisplatin treatment period. 130 (34.3%) patients had otologic complaints following cisplatin treatment. Audiograms were ordered for 20 (7.8%), 13 (5.1%), and 16 (6.2%) patients at 1-month, 3-month, and 6-month follow-ups, respectively. No patients in the study cohort received baseline, treatment, and post-treatment audiograms as recommended by national ototoxicity monitoring protocols. Patients with Head and Neck Cancer (HNC) represented the largest subgroup that received cisplatin (*n* = 122, 32.2%) and demonstrated higher rates of ototoxicity counseling (*n* = 103, 84.4%) and pretreatment audiograms (*n* = 70, 57.4%) compared to the non HNC group (*n* = 36, 36.2%, *P* < 0.0001 and *n* = 22, 8.5%, *P* < 0.0001).

**Conclusions:**

There is poor adherence to national ototoxicity monitoring guidelines at a large academic medical center. This is a missed opportunity for intervention and aural rehabilitation. Improved education and collaboration between otolaryngology, audiology, and medical oncology is needed to develop and promote an effective ototoxicity-monitoring program.

**Graphical abstract:**

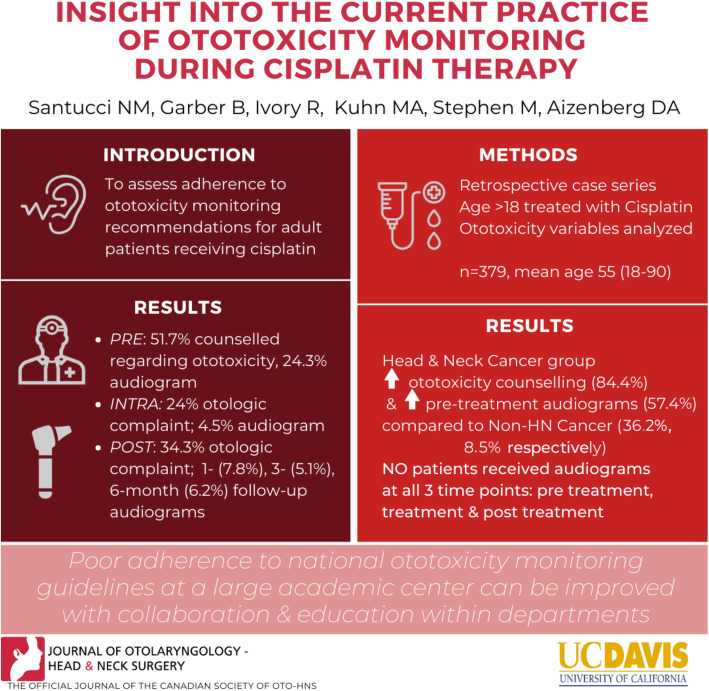

## Introduction

Platinum-based agents are standard curative and palliative chemotherapies used for a wide range of malignancies in both the pediatric and adult population. Ototoxicity, the hearing disorder that results from temporary or permanent inner ear dysfunction after treatment with an ototoxic drug, is a well-documented side effect of platinum-based agents. Cisplatin is considered one of the most ototoxic pharmacologic agents, typically causing bilateral high frequency sensorineural hearing loss with progression to lower frequencies with continued exposure. The potential for permanent bilateral sensorineural hearing loss and tinnitus can occur both during treatment and up to 136 months after therapy completion [1–8] with an incidence of 20–84% [9, 10].

The hearing loss caused by cisplatin is due to degeneration of the cochlear hair cells, supporting cells, spiral ganglion cells and marginal cells of the stria vascularis. The outer hair cells are damaged before the inner hair cells and cisplatin damage occurs in a relatively orderly manner from base (high frequency) to apex (low frequency) destruction. A main mechanism of cisplatin ototoxicity is associated with the production of free radicals [5, 6, 11–13]. The degree of hearing loss has been associated with cumulative dosing of platinum agents, duration of treatment, concurrent treatment with radiotherapy, history of noise exposure, and method of administrations (bolus versus infusion) [1, 3, 6, 9–15]. Evidence also suggests a genetic component to the risk factors for cisplatin-induced ototoxicity, with an estimated 38–47% [16] of individual variability linked to polymorphisms in genes encoding DNA repair enzymes and membrane pumps [11–13, 16, 17]*.*

Hearing loss has considerable quality of life implications, with notable effects on a patient’s social and emotional needs [4, 6, 18, 19]. These impacts include safety concerns [6], communication ability [4, 6, 18], increased rates of depression, anxiety and social isolation [4, 6, 10, 18], and higher incidence of hospital admission [20], compounding what is already experienced by patients diagnosed with a critical illness [4, 10]. Ototoxicity monitoring programs can to a large extent avert the reduced quality of life as a result of hearing loss, since at-risk patients can be identified early, counselled, monitored and managed appropriately through a logical and systematic way [5].

Ototoxicity monitoring guidelines including those from the American Speech and Hearing Association (ASHA), and American Academy of Audiology (AAA) [21], outline an idealistic approach for ototoxicity monitoring programs [6, 10]. ASHA and AAA Guidelines, established in 1994 and 2009 respectively, provide a set of broad goals for monitoring for ototoxicity including performing pretreatment baseline evaluation and counseling, monitoring visits at intervals to document hearing loss progression and follow up evaluations to determine post treatment effects. Audiometric criteria defining an ototoxic hearing shift are defined with guidelines outlining the grades of ototoxic change. Ototoxicity typically begins in the frequencies above 8000 Hz and progresses to lower speech frequencies. Therefore, ASHA and AAA recommend that baseline assessment should include behavioral measures such as pure-tone audiometry (PTA) from 250 Hz to 8000 Hz and high-frequency audiometry (HFA) from 9000 Hz to 20,000 Hz, plus objective measures such as distortion product of otoacoustic emissions (DPOAEs) and tympanometry, along with self-evaluating questionnaires [22].

Recent concerns have been raised that ototoxicity monitoring is an inconsistent practice and barriers to implementation of a monitoring program include failure to enroll patients in the program, patients lost to follow-up, and baseline and monitoring tests not being conducted within the pertinent time windows [4]. Konrad-Martin et al. investigated five ototoxicity monitoring programs (OMPs), outlining how patients in each OMP were identified and followed and specific barriers to effective monitoring in each program. Effective ototoxicity monitoring guidelines are essential as there are no known pharmacologic preventive or treatment strategies for cisplatin-induced ototoxicity that are effective without diminishing the antineoplastic efficacy of the drug [11]. To better assess adherence to available ototoxicity monitoring recommendations, the current state of ototoxicity monitoring for adult patients receiving cisplatin at a large academic institution was evaluated.

## Material and methods

This study was approved by the UC Davis Institutional Review Board. We reviewed the medical records of adult patients (> 18) identified by the UC Davis pharmacy as having received cisplatin between January 2014 and August 2017. Demographic and clinical data collected included age, gender, type of malignancy being treated, curative versus palliative treatment goal, number of intended treatments, cumulative dose of cisplatin and history of alcohol and tobacco abuse, prior head and neck radiation, and hearing loss prior to treatment. Additional clinical characteristics collected included ototoxicity counselling received prior to treatment, pretreatment audiogram, otology complaints during and after treatment, and audiology monitoring and Otolaryngology and/or Audiology referrals obtained during treatment and after completion of treatment. Audiometric testing was defined as any patient who received conventional or extended high frequency audiometry or otoacoustic emissions.

Patient audiograms were analyzed with an adult grading scale from the National Cancer Institute (NCI) common terminology criteria for adverse events [23] . Of note, in order to use the NCI common terminology criteria for adverse events grading scale, a baseline audiogram is needed.

Summary analysis for the study cohort was performed. Differences in ototoxicity monitoring between patients receiving cisplatin for a head and neck malignancy versus non-head and neck malignancy were compared using Pearson chi-squared and Fisher exact tests. A significance value of *p* < 0.01 was used.

## Results

### Characteristics of cohort

We reviewed 485 patient medical records. One hundred and seven patients were excluded because of the following criteria: Patients had a transfer of care during treatment, less than 3 months of follow up after treatment completion, or death during or within 3 months after treatment.

Three hundred seventy-nine patients met study criteria with a mean age of 55 years (range 18–90) at the beginning of treatment. 60% of patients identified as male and 40% as female. Patients were treated for a wide range of malignancies with the majority being head and neck (122), gynecologic (74), urologic (70), and lung (51). Treatment was with curative intent in most patients, with a small subset undergoing palliative chemotherapy (17.2*%).* Sixteen patients (4.2%) had a known history of prior radiation to the head and neck and 204 (53.8%) patients received concurrent radiation treatment. 102 (27.4%) patients had a history of hearing loss documented in the medical record prior to treatment. (Table 1*: Patient Demographics and Clinical Characteristics*).

**Table 1:** Patient Demographics and Clinical Characteristics

Characteristic	N (%)
Sex	
Male	216 (60)
Female	163 (40)
Malignancy type	
Breast	3 (0.8)
Central Nervous System	12 (3.2)
Gastrointestinal	32 (8.4)
Gynecologic	74 (19.5)
Head & Neck	122 (32.2)
Hematologic	3 (0.8)
Lung	51 (13.5)
Sarcoma	7 (1.8)
Skin	5 (1.3)
Urologic	70 (18.5)
Treatment intent	
Curative	305 (80.5)
Palliative	65 (17.2)
Unknown	9 (2.3)
History of Prior Radiation to Head and Neck	
Yes	16 (4.2)
No	312 (82.3)
Unknown	51 (13.5)
Concurrent Radiation Treatment	
Yes	204 (53.8)
No	175 (46.2)
History of Hearing Loss prior to treatment	
Yes	104 (27.4)
No	183 (48.3)
Unknown	92 (24.3)

### Ototoxicity monitoring

Prior to initiation of cisplatin chemotherapy, 51.7% of patients had documented counseling regarding the ototoxic nature of the agent. In the pre-treatment period, an audiogram was ordered for 92 (24.2%) patients, with 83 (90.2%) performed. Seventy-three of the 92 (79.3%) audiograms ordered were indicated to be for the purpose of ototoxicity monitoring (Table 2).

**Table 2:** Current State of Ototoxicity Monitoring

		All Malignancies		
	All malignancies (*n* = 379)	Head and Neck (*n* = 122)	Non-Head and Neck (*n* = 257)	
	N (%)	N (%)	N (%)	*P* value*
**Prior to treatment with cisplatin**				
Counselled about ototoxicity risk	196 (51.7)	103 (84.4)	93 (36.2)	***P*** **< .0001**
Baseline pre-treatment audiogram ordered	92 (24.3)	70 (57.4)	22 (8.5)	***P*** **< .0001**
**During cisplatin treatment**				
Otologic Complaints	91 (24)	51 (41.8)	43 (16.7)	***P*** **< .0001**
Treatment changed following otologic complaints	25 (27.5)	15 (29.4)	10 (23.2)	*P* = .5010
Audiogram ordered during treatment	17 (4.5)	6 (5.0)	11 (4.3)	**P = .7937
**Following cisplatin treatment**				
Otologic Complaints (total)	130 (34.2)	75 (61.5)	55 (40)	***P*** **< .0001**
1-month post-treatment audiogram	20 (7.8)	13 (10.6)	7 (2.7)	**P **= .0024**
3-month post-treatment audiogram	13 (5.1)	11 (9.0)	2 (0.8)	**P **= .0001**
6-month post-treatment audiogram	16 (6.2)	12 (9.8)	4 (1.6)	**P **= .0004**
Audiogram or Otolaryngology Consult 6–24 months post treatment	38 (15)	22 (18)	16 (6.2)	***P*** **= .0003**

*P value used to compare Head and Neck Cancer and Non-Head and Neck Cancer Groups. Significance level set at *p* < .01, and results are bolded if statistically significant

**Fisher exact test statistic value

During the cisplatin treatment period, 91 (24%) patients had documented otologic complaints including hearing loss (*n* = 25), tinnitus (*n* = 38), or both (*n* = 28). In patients with otologic complaints, 66 (72.5%) had no change in their treatment regimen. 13 (14.3%) patients received a change in dose, 6 (6.6%) had a change in drug, and chemotherapy was terminated in 6 (6.6%) patients. During the treatment period, only 15 (16.4%) patients were referred for audiogram in response to subjective otologic complaints.

Using available audiograms, ototoxicity was graded using the NCI common terminology criteria for adverse events [23], 3 of the 15 (20%) patients with subjective hearing complaints during the cisplatin treatment period met criteria for an ototoxic hearing change (2 Grade II, 1 Grade III). For 4 patients, there was no baseline audiogram for comparison therefore grading of hearing loss was not performed. Two patients were referred to an Otolaryngologist for further evaluation.

At 1-month post-treatment, 130 (34.3%) patients had documented otologic complaints of hearing loss (*n* = 50), tinnitus (*n* = 34), or both (*n* = 46). Of these 130 patients, 19 (14.6%) were referred for audiogram and 12 (9.2%) were performed. Of these 12 patients, 7 (58.3%) showed an ototoxic change (2 Grade II, 5 Grade III), 1 had no ototoxic change, and 4 patients had no baseline audiogram available. 42% of patients who had an audiogram ordered were referred to the otolaryngology department. A consult for hearing aids or other additional intervention was recommended for 7 of the 19 patients who received an audiogram at the 1-month follow-up. Only 1 patient of the 249 patients without otologic complaints received an audiogram at 1-month follow-up for monitoring purposes.

At 3-month and 6-month follow-up, 13 and 16 audiograms were ordered, respectively. Of the 112 patients that were followed for a duration of 24 months or longer, 32 patients (28.6%) received at least 1 post-treatment audiogram.

No patients in the study cohort underwent baseline, during treatment, and post-treatment audiograms as recommended in both the ASHA and AAA ototoxicity monitoring protocols.

### Ototoxicity monitoring by malignancy type

Of all cancer sites receiving cisplatin, patients with head and neck cancer (HNC) represented the largest subgroup, 122 of the 379 patients (32.2%). Patients with HNC had higher rates of ototoxicity counselling (84.4% versus 36.2%) and pretreatment audiograms (57.4% versus 8.6%) compared to patients with non-head and neck malignancies (*P* < .0001). Overall, patients with gynecologic malignancies (*n* = 74) had the lowest rates of documented counseling regarding the ototoxic risks of cisplatin chemotherapy (16.2%, *p* < 0.0001) and none of these patients received pretreatment audiograms. HNC patients also had more documented otologic complaints during (41.8% versus 16.7%) and following (61.2% versus 21.4%) cisplatin treatment period (*P* < .0001). 6 of the 17 patients (35.3%) who were referred for an audiogram during treatment were being treated for HNC.

## Discussion

According to the American Cancer Society, an estimated 1.8 million people in the United States will be newly diagnosed with cancer this year and most will live following their diagnosis and treatment (American Cancer Society, 2020). The 5-year survival rate for all cancers in the U.S. is 67.4% (2010–2016) [24]. Platinum-based drugs are the chemotherapeutic agents of choice for the treatment of many cancers; approximately half of all patients undergoing chemotherapeutic treatment receive a platinum drug [25]. Ototoxic side effects of cisplatin chemotherapy, including hearing loss and tinnitus, can have profound consequences on a patient’s social, educational, and vocational status. An effective ototoxicity monitoring program detects cochlear injury prior to a patient subjectively reporting hearing loss or quality of life detriments, allowing potential intervention to prevent the progression of inner ear damage.

In 1994, the American Speech and Language Hearing Association (ASHA) issued national ototoxicity monitoring guidelines for patients receiving potentially ototoxic treatments [26]. The American Academy of Audiology (AAA) issued a position statement and clinical practice guidelines in 2009 regarding the necessity and implementation of ototoxicity monitoring [21]. Recommendations from both professional organizations include comprehensive baseline testing, follow up evaluations prior to cycles of platinum-based chemotherapy and follow up audiometry after completion of treatment. Despite the established clinical practice guidelines, anecdotal evidence suggests that adherence to such recommendations is poor.

The options for reducing ototoxicity include reducing the drug dose or switching to a less ototoxic agent. Several clinical trials have demonstrated that lower dose cisplatin regimens may have a significant impact in reducing toxicity while maintaining efficacy in certain subsets of patients [13]. Other studies have demonstrated a change of cisplatin to carboplatin in combination with radiotherapy to avoid serious toxicities such as renal toxicity, and neurotoxicity has led to positive results with regards to patient outcome or overall survival [1]. Further studies are needed to examine ototoxicity and survival outcomes with any change in current treatment regimen. In addition, much work is currently being put into finding ways to protect the inner ear from the ototoxic effects of platinum-based agents. Although some results are promising, no agent is currently recommended for routine use.

Routine monitoring with audiograms for early detection of changes in hearing status inform adjustments to treatment plans, initiation of aural rehabilitation, and mitigation to impact on patient quality of life [4]. To our knowledge, this study is the first to evaluate ototoxicity monitoring practices in one large academic institution. The findings reflect very poor adherence to ototoxicity monitoring recommendations as compared to ASHA and AAA clinical practice guidelines.

There are differences in ototoxicity monitoring in HNC patients compared to those with other malignancies. These included more pretreatment ototoxicity counseling and increased pretreatment audiograms. It’s plausible that these higher rates were observed in patients seen by Otolaryngologists who keenly understand ototoxicity risk and the impacts of sensorineural hearing loss. Of note, the patients with HNC also had the greatest number of documented otologic complaints during and after treatment. Such patients are at greater risk for otologic toxicity, given the proximity of the malignancy to the auditory system, and therefore side effects from surgery and radiation, with the cochlea being an exquisitely radiosensitive organ [27]. Furthermore, head and neck surgeons, and potentially head and neck oncologists, are more accustomed and possibly more likely to inquire about and act upon otologic symptoms. Regardless, while HNC patients received better ototoxicity monitoring, patterns were still very poor.

In view of advances in early cancer detection, supportive care and treatment, there are more than 13.7 million cancer survivors, comprising 4% of the United States population with the number expected to increase 2% annually [28, 29]. For many patients these marked improvements in survival have been countered by serious therapy adverse effects. These include sequalae such as permanent hearing loss that can impair functional status, workplace productivity and overall quality of life. Hearing aids are often the initial intervention for hearing loss in the setting of ototoxicity and primarily allow for sound amplification [30, 31]. Difficulties with voice discrimination, particularly in noisy environments is common amongst hearing aid users as normal hearing cannot be restored [31]. Other limitations of hearing aids include affordability and the stigma associated with their use [30]. It should also be acknowledged that audiologic rehabilitation may not the predominant priority for patients with an oncologic history given required follow-up for recurrence surveillance and additional sequela. These findings highlight opportunities to improve monitoring and interventions for cancer patients receiving potentially ototoxic chemotherapy.

A successful ototoxicity monitoring program involves a multidisciplinary effort of oncology, audiology, pharmacy, and otolaryngology that is integrated into patient care pathways [4]. Clear communication and documentation as well as standardized timelines for screening are critical. Possible improvements include implementation of a system-wide ototoxicity monitoring program using built-in ordering prompts for ototoxic medications and using portable audiogram technologies to reduce the burden of additional testing appointments in the audiology office. A recent model that can predict posttreatment hearing loss prior to radiotherapy and cisplatin treatment initiation in patients with HNC (sensitivity 80%, specificity 75%) may also support in counseling patients of the risks of therapy [32]. Furthermore, wide-spread auditing of adherence to ototoxicity monitoring guidelines is important for establishing improved patterns of care. Until new otoprotective cancer therapies are established and the incidence of ototoxicity can be reduced, harm minimization via effective monitoring and rehabilitation must be optimized.

This study does have several limitations; Only patients receiving cisplatin were included in the study population despite other well-known commonly prescribed ototoxic agents including other platinum based antineoplastic drugs and certain aminoglycosides. Furthermore, patients under the age of 18 were not included in this study thus limiting generalizability of findings to ototoxicity monitoring in the adult population. Given data was obtained through a retrospective medical chart review, poor documentation can introduce bias into the results. Finally, this study included patients from a single academic medical center, which may not be representative of national trends in ototoxicity monitoring. However, given these finding we would encourage other academic medical centers to assess their own ototoxicity monitoring protocols. Further studies will be needed to investigate otolaryngologist’s attitudes, knowledge, and perception of ototoxicity monitoring as well as perceived adherence to ototoxicity monitoring programs at their respective institution.

## Conclusion

Despite well-established ototoxicity monitoring guidelines, it seems that clinical practice may not reflect these recommendations. However, there is minimal data detailing actual ototoxicity monitoring in patients receiving platinum chemotherapeutics. The present findings suggest poor adherence to national recommendations in a large academic center. They underscore the need for improved education and collaboration between disciplines to develop and promote an effective ototoxicity-monitoring program. Expeditious identification of ototoxicity holds for potential for improved interventions, aural rehabilitation, and quality of life in patients with critical illnesses.

## Data Availability

The data generated and analyzed during the study are available from the corresponding author on reasonable request.

## Abbreviations

AAA: American Academy of Audiology
ASHA: American Speech and Hearing Association
DPOAEs: Distortion product of otoacoustic emissions
HFA: High-frequency audiometry
HNC: Head and Neck Cancer
NCI: National Cancer Institute
UC: University of California
